# Supplementary material for: Relationship between the asymmetry of the resting scapular position and the prevalence of latent myofascial trigger points in the trapezius muscle in asymptomatic adults
Source: PLoS One. 2025 Oct 27;20(10):e0335268. doi: 10.1371/journal.pone.0335268 (PMC12558538; doi:10.1371/journal.pone.0335268)
Supplement: S1 Table — (DOCX) [file pone.0335268.s001.docx]

**Supplemental Table 1.** Characteristics of the participants and asymmetry of the resting scapular position of the two groups, which were divided based on the presence of latent myofascial trigger points in the left UTM.

|  | **No L-MTrP (n = 19)** | **L-MTrPs (n = 13)** | **p** |
| --- | --- | --- | --- |
| Age (year) | 25.11 ± 1.11 | 28.00 ± 2.08 | >0.05^a^ |
| Height (cm) | 170.84 ± 0.95 | 168.62 ± 0.91 | >0.05^b^ |
| Weight (kg) | 68.26 ± 2.09 | 69.85 ± 2.11 | >0.05^b^ |
| Dominant arm (R/L) | 17/2 | 10/3 | >0.05^c^ |
| HSAA (°) | 0.16 ± 0.73 | −2.11 ± 0.80 | >0.05^b^ |

HSAA, horizontal scapular alignment angle; L-MTrPs, latent myofascial trigger points.

^a^Assessed using the Mann–Whitney U test.

^b^Assessed using Student’s t-test.

^c^Assessed using the chi-square test.
