# Supplementary material for: Relationship between the asymmetry of the resting scapular position and the prevalence of latent myofascial trigger points in the trapezius muscle in asymptomatic adults
Source: PLoS One. 2025 Oct 27;20(10):e0335268. doi: 10.1371/journal.pone.0335268 (PMC12558538; doi:10.1371/journal.pone.0335268)
Supplement: Attachment — Submitted filename: Response to Reviewers.docx [file pone.0335268.s003.docx]

**Reviewer #1, Comment 1:**

Diagnostic Method of Latent MTrPs

The manuscript does not clearly specify whether latent MTrPs were identified through palpation or ultrasound. Please detail the diagnostic criteria (e.g., Travell and Simons’ standards), techniques used, number of examiners, and any reliability checks. This is essential for methodological rigor.

**Response:**

To address this comment, we rewrote this section and added new information to the Methods and References sections, as stated below. Detailed information regarding the diagnostic criteria used here is also provided in descriptions of other techniques used to identify latent MTrPs.

A single assessor with 10 years of clinical experience identified latent MTrPs in the right and left UTMs by manual palpation. The diagnostic criterion for latent MTrPs was the presence of hypersensitivity tender points in the muscle taut band [3]. This procedure involved two steps: First, a palpable taut band was located via flat (cross-fiber) palpation. In this procedure, the assessor gently pushed their thumb pad inward, then slid it perpendicular to the muscle fibers. Second, the assessor systematically palpated within the taut band to identify potentially hypersensitive areas, then applied sustained and gradually increasing pressure for 3–5 seconds to confirm heightened sensitivity. The subject was asked to explicitly confirm that they experienced pain, and their response was compared to the reaction elicited by applying pressure to adjacent non-tender areas within the same muscle. We note that the inter-rater reliability when identifying MTrPs in the upper quarter muscles, including the upper trapezius muscle, has been reported to be high when assessed by experienced examiners [31].

31. del Moral OM, Lacomba MT, Russell IJ, Méndez ÓS, Sánchez BS. Validity and reliability of clinical examination in the diagnosis of myofascial pain syndrome and myofascial trigger points in upper quarter muscles. Pain Medicine. 2018;19:2039-2050. doi: 10.1093/pm/pnx315.

**Reviewer #1, Comment 2:**

Update and Strengthen Literature

The introduction relies heavily on older sources. To strengthen the rationale and ensure alignment with current science, please incorporate recent literature on myofascial dysfunction and postural relationships, such as:

<https://doi.org/10.1016/j.jbmt.2023.12.005>

<https://doi.org/10.1186/s13102-023-00719-y>

<https://doi.org/10.3390/s24030718>

<https://doi.org/10.3389/fspor.2024.1412412>

**Response:**

In response to this comment, we have rewritten the introduction and cited more recent literature, as outlined below.

The mechanism of MTrP formation has been proposed to involve mechanical stress from repetitive or sustained movements, which overload the muscle and result in sustained, localized muscle contractions (muscle taut bands).

1. Lam C, Francio VT, Gustafson K, Carroll M, York A, Chadwick AL. Myofascial pain - A major player in musculoskeletal pain. Best practice & research. Best Pract Res Clin Rheumatol. 2024;38:101944. doi: 10.1016/j.berh.2024.101944.

2. Shah JP, Thaker N, Heimur J, Aredo JV, Sikdar S, Gerber, L. Myofascial trigger points then and now: A historical and scientific perspective. PM R. 2015;7:746-761. doi: 10.1016/j.pmrj.2015.01.024.

7. Edwards J. The importance of postural habits in perpetuating myofascial trigger point pain. Acupunct Med. 2005;23:77-82. doi: 10.1136/aim.23.2.77.

8. Steen JP, Jaiswal KS, Kumbhare D. Myofascial pain syndrome: An update on clinical characteristics, etiopathogenesis, diagnosis, and treatment. Muscle Nerve. 2025;71:889-910. doi: 10.1002/mus.28377.

**Reviewer #1, Comment 3:**

**Discussion Formatting**

Subheadings in the discussion section disrupt the flow. Please remove them and present the discussion as a cohesive narrative in line with PLOS ONE’s style.

**Response:**

As per the reviewer recommendation, we have removed all subheadings in the Discussion section.

**Reviewer #1, Comment 4:**

Revise Line 47 for grammatical accuracy: “Simons explained…” rather than “They explained…”.

**Response:**

Based on other comments regarding of nature of the scientific literature, this line has been rewritten as follows: The mechanism of MTrP formation has been proposed to involve mechanical stress from repetitive or sustained movements, which overload the muscle and result in sustained, localized muscle contractions (muscle taut bands) [1-3].

**Reviewer #1, Comment 5:**

In Figure 2 caption, replace “patients” with “participants” to reflect the healthy cohort.

**Response:**

In response to this comment, we have corrected “patients” to “participants”.

**Reviewer #1, Comment 6:**

Clarify in Methods/Ethics whether participants were screened for physical activity levels or dominant-side training, as these factors may influence scapular position.

**Response:**

According to the comment, we add the information to the methods as stated below:

No specific exclusion criteria were applied based on physical activity levels or dominant-side sport-specific training.

Additionally, we add the information to the discussion as stated below:

Third, this study did not screen participants for physical activity level or having a history of dominant-side training. Either factor could potentially influence scapular resting position and kinematics. Therefore, future studies should consider controlling for these variables. For example, this could involve recruiting participants from specific athletic populations and/or by quantifying participant activity levels.

**Reviewer #1, Comment 7:**

Conduct a thorough language and style edit, ideally by a native English speaker with biomedical expertise, to improve clarity and polish.

**Response:**

Thank you for your valuable suggestion. We have conducted a thorough language and style revision of the entire manuscript with the assistance of a native English speaker with expertise in biomedical writing. We have carefully edited the text to improve clarity, grammar, and overall readability. All changes have been incorporated in the revised version of the manuscript.

**Reviewer #2, Comment 1:**

To enhance the educational impact, I recommend including a figure or diagram that transparently overlays the anatomical reference points of the scapula on the photo provided. This would help readers and clinicians better visualize the relationship between scapular alignment and trigger point localization.

**Response:**

As suggested in this comment, we have included a revised Figure in the latest version of our manuscript. We have also rewritten the Figure Legend as stated below:

Key anatomical landmarks of the scapula are depicted in the photograph. The dashed line indicates the (virtual) contour of the scapula. MSS: Medial spine of the scapula, AA: Acromial angle, SAS: Superior angle of scapula, IAS: Inferior angle of scapula.
